# Antihyperglycemic effect of *Vernonia amygdalina* and in vitro evaluation of its antiproliferative activity on human osteosarcoma MG-63

**DOI:** 10.11604/pamj.2022.42.222.33149

**Published:** 2022-07-21

**Authors:** Barnabé Lucien Nkono Ya Nkono, Ablassé Rouamba, Ioana Alexandra Duceac, Liliana Verestiuc

**Affiliations:** 1 Department of Biological Sciences, Higher Teacher Training College, University of Yaounde 1, Yaounde, Cameroon,; 2 Laboratoire de Biochimie et Chimie Appliquées, Université Joseph Ki-Zerbo, Ouagadougou, Burkina Faso,; 3 Faculty of Medical Bioengineering, Grigore T. Popa University of Medicine and Pharmacy, Iasi, Romania

**Keywords:** *Vernonia amygdalina*, antihyperglycemic, antiproliferative, human osteosarcoma MG-63, rat

## Abstract

**Introduction:**

the leaves of Vernonia amygdalina (V. amygdalina) are consumed as food in sub-Saharan Africa (SSA). In traditional medicine, this plant is widely used in the treatment of cancer and diabetes mellitus. In the present study, we evaluated the antihyperglycemic and the antiproliferative activities of the hydroalcoholic extract of V. amygdalina leaves (HAEVa).

**Methods:**

we conducted an experimental descriptive and analytical study with a prospective data collection from May 2019 to July 2020. For the in vivo study, the experiments were carried out on albino male rats of Wistar strain (Rattus norvegicus). Antihyperglycemic activity was performed in vivo in dexamethasone-induced insulin-resistant rats using the oral glucose tolerance test (OGTT). The biocompatibility and the antiproliferative activity of extract were performed in vitro respectively on rabbit primary dermal fibroblasts (RPDF) and human osteosarcoma MG-63 cells using the 3-(4,5-dimethylthiazol-2yl)-2,5-diphenyltetrazolium bromide (MTT) assay. The data were analyzed with the GraphPad Prism software version 5.0.3. The statistical analyses were obtained by the analysis of variance (ANOVA), followed by Bonferroni´s post-test. P<0.05 was considered as the minimal level of statistical significance.

**Results:**

regarding to the antiproliferative investigation, extract at 125, 250 μg/mL exhibited a significant cytotoxic effect on human osteosarcoma MG-63 compared to the vehicle (p<0.001) in a dose-response manner after 24h, 48h of exposure to HAEVa. Interestingly, HAEVa in concentrations of 125 and 250μg/ml showed no cytotoxicity (p>0.05) on RPDF after the different times of exposure. However, HAEVa in a high concentration of 500 μg/mL wasn´t biocompatible with RPDF. HAEVa also prevented postprandial blood glucose level in dexamethasone-induced insulin-resistant rats at both doses tested (p>0.05 and p<0.01 at doses of 50 and 100 mg/kg respectively).

**Conclusion:**

the results of this study suggest that HAEVa has antiproliferative properties on MG-63 osteosarcoma in vitro and also inhibits in vivo the postprandial blood glucose level in dexamethasone-induced insulin-resistant rats.

## Introduction

Noncommunicable diseases (NCDs) currently cause more deaths than all other causes combined and NCD deaths are projected to increase from 38 million in 2012 to 52 million by 2030 [1]. Moreover, it is apparent from that global status report that four major NCDs such as cardiovascular diseases, cancer, chronic respiratory diseases and diabetes are responsible for 82% of NCD deaths. The global burden of disease study estimated a 67% increase in disability-adjusted life-years attributable to NCDs in SSA from 190 to 2017, with the burden of cancer (79.5%) and diabetes (83.1%) exhibiting the largest relative increases over this period [2]. Therefore, building on the 1978 Alma-Ata Declaration on primary health care, WHO encourages the governments of African countries and specifically those of sub-Saharan Africa to vulgarize the results that have scientifically validated the empirical use of certain plants used in the traditional pharmacopoeia to address the primary health problems of its low-income population whose access to conventional medicines is generally out of reach [3].

**Figure 1:**
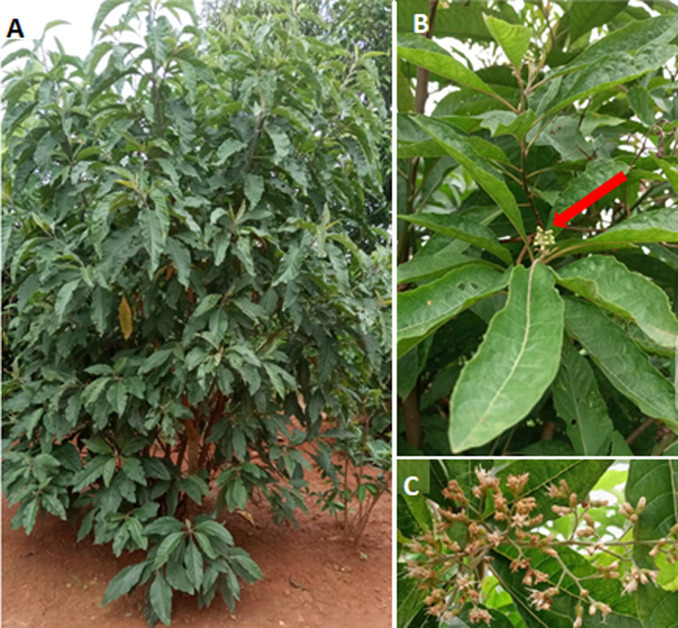
*Vernonia amygdalina*, A) whole plant; B) flower bud; C) floral bouquet

*Vernonia amygdalina* is a plant that generally grows wild in most tropical African countries (Figure 1 A). The leaves (Figure 1 B), usually have regular bisexual flowers (Figure 1 C) are generally consumed as a food in sub-Saharan Africa and particularly in Cameroon and Nigeria as a vegetable. *V. amygdalina* is known by various names in SSA. The plant is known as “kongo bololo” in the Democratic Republic of Congo, “ndolé” in Cameroon, “awonoo” in Ghana and “kougôpô” or “anango” in Côte d'Ivoire. In traditional medicine, this plant is widely used in the treatment of cancer and diabetes [4-6]. It has been scientifically shown that the aqueous extract of the leaves of *V. amygdalina* has hypoglycemic properties in alloxane-induced diabetic albino Wistar rats with similar potent to those of glibenclamide, a reference antidiabetic drug [7]. It was also demonstrated the synergistic effect of the aqueous extract of *V. amygdalina* with the oral antidiabetic metformin in the potentiation of the hypoglycemic effect in normoglycemic Wistar rats [8]. Furthermore, *V. amygdalina* extract has anti-cancer activities in human breast cancer cell lines [9].

However, there is a lack of information on the hypoglycemic, antihyperglycemic and antiproliferative activities of this plant on experimental models of type 2 diabetes mellitus as well as on osteosarcoma cells. Previously conducted studies have not addressed these pharmacological activities of *V. amygdalina* on experimental models of type 2 diabetes mellitus as well as the osteoblast-like human cell line (MG-63).

The purpose of this study was to evaluate the antihyperglycemic activity of the hydroalcoholic extract of *V. amygdalina* leaves (HAEVa) in dexamethasone-induced insulin resistant albino Wistar rat. In addition, this study was also conducted to evaluate the antiproliferative effect of HAEVa on MG-63 human osteosarcoma.

## Methods

**Study design***:* an experimental descriptive and analytical study with a prospective data collection from May 2019 to July 2020 was carried out in vivo and in vitro with the aim of evaluating the antihyperglycemic and antiproliferative activity of HAEVa. The study of the antihyperglycemic activity of HAEVa was carried out in vivo using dexamethasone-induced insulin-resistant rat model while the antiproliferative activity of HAEVa was carried out in vitro on human osteosarcoma MG-63 cells.

**Study setting and population plant materials:** the leaves of *V. amygdalina* (Figure 1 B) were collected from a private farm (latitude 3°52'0.001''North, longitude 11°31'0.001 East, altitude above sea level 726m, humidity 73.13% in dry season and 88.54% in rainy season, annual precipitation level 1727mm) with the owner´s permission and identified in the national herbarium in comparison with specimen No. 25625/SRF/Cam which was already part of the collection of the national herbarium. The fresh leaves were dried for 10 days at room temperature in the laboratory. Once dried, the leaves were crushed using an electric grinder and then sieved using a sieve of 0.5 mm mesh. The preparation of HAEVa was done by maceration in a water-ethanol mixture (30: 70) at a rate of 200 g/l. The mixture was macerated at room temperature away from light for 24 hours and then filtered using whatman paper No 4. The filtrate was concentrated with rotavapor at 45°C until complete exhaustion of the solvent. The anhydrous hydroalcoholic extract of V. amygdalina was stored in a dark glass jar for later use.

**Experimental animals:** the study was carried out on albino male rats of Wistar strain (*Rattus norvegicus*), eight weeks old at the beginning of the experiment.

**Induction of hyperglycemia:** hyperglycemia was induced by daily injection of dexamethasone (RYAN PHARMA®-UK) at a dose of 5 mg/kg subcutaneously once daily following the method described by Kumar *et al*. [10].

**Experimental design:** dexamethasone-induced insulin-resistant model were divided into four groups of five animals each for the experiment: group I: insulin-resistant negative control (IRNC) group, fed with deionized water (10 ml/kg) for fourteenth days; group II: insulin-resistant positive control group, treated with glibenclamide at daily dose of 5 mg/kg for fourteenth days; groups III and IV: insulin-resistant treated groups, fed daily with HAEVa at doses of 50 and 100 mg/kg respectively for fourteenth days. The same distribution of animal groups was also made for experimentation in normoglycemic rats. Group I: normal water control group (NWC), group II: normal glibenclamide positive control group and groups III and IV: normal treated groups with HAEVa.

**Figure 2:**
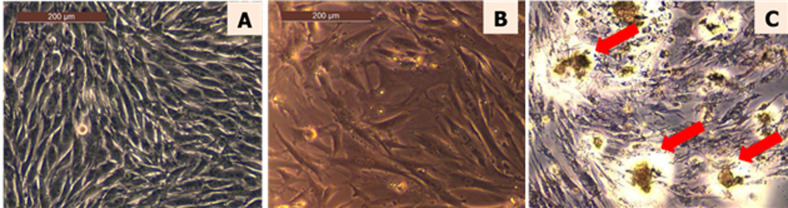
cell cultures, A) osteoblasts MG-63 in culture flask, after 5 days; B) fibroblasts 72 h (48 well plate); C) formazan crystals in fibroblasts after 72 h of direct contact with a composite material (arrows)

**Cells culture:** cell culture was performed using the method described by Tanase *et al*. [11]. Osteoblasts (Figure 2 A) or primary dermal fibroblasts (Figure 2 B) on passage 6 were cultured in Dulbecco's Modified Eagle Medium (DMEM) supplemented with 10% fetal bovine serum (FBS) and 1% penicillin-neomycin-streptomycin (P/N/S), in a humidified atmosphere of 5% CO_2_ at 37°C. When the cells reached sub-confluence, they were trypsinized with 0.25% trypsin containing 1 mM ethylenediamine tetraacetic acid (EDTA) and transferred on 96-well plates.

**Variables:** for in vitro tests, the variables considered were fasting blood glucose level and postprandial blood glucose level in normoglycemic rats and dexamethasone-induced insulin-resistant rats at 0 min and 30, 60, 90 and 120 minutes respectively after oral glucose overload. On the other hand, for in vitro tests, only one variable was considered, namely cell viability after 24, 48 and 72 hours of exposure of cells to HAEVa.

**Bias:** after 7 days of dexamethasone injection, animals that had a fasting blood glucose level greater than 110mg/dl after a minimum fast of 12 hours were considered as insulin-resistant and used for our experiments. Animals with a blood glucose level below 110mg/dl were excluded from the experiment. For in vitro testing, cell cultures that had less than 3000 cells per ELISA microplate well were excluded. Only wells containing at least 3000 cells were selected for the experiments.

### Data resource and measurement

**Oral glucose tolerance test:** the OGTT was performed as previously described [12]. After fourteen days of administering the treatments to the different groups of insulin-resistant animals, they were subjected to 12 hours fasting before performing the oral glucose tolerance test (OGTT). OGTT was also performed in normoglycemic animals that received a single dose of HAEVa as well as different treatments. After measuring the fasting blood glucose levels of the different groups using a viva check ino brand glucometer® using blood samples obtained from the puncture of the rats' tails, the treatments were immediately administered to the animals. Thirty minutes after administration of the last treatment dose, all groups received a single dose of D-glucose (3 g/kg b.w) by oral gavage and blood glucose levels were then taken at 30, 60, 90 and 120 min after glucose administration. The percentage of variation of glycemia was expressed as calculated by equation (1). Each result represents the mean ± standard error mean (SEM) of five independent experiments:


$$Blood\text{ glucoe level (\%)=}\frac{Glycemia\text{ of samples}}{Glycemia\text{ of control}}\times 100\ldots \;\ldots \;\ldots \text{(1)}$$

**MTT viability assay:** the extract was solubilized according to the method previously described by Rouamba *et al*. [13]. HAEVa was sterilized by filtration through a 0.22 ®m diameter microporous filter to be further tested using a standard MTT assay, according to ISO 10993-5. Three parallel samples from each extract concentration were put in contact with the cell culture in the wells. For reference purposes, cells were seeded under the same condition. After 24, 48 and 72 hours of incubation, the medium was aspirated, 100 ®L of MTT solution (0.5%) was added and the plate was incubated for 3 h. After the exposure, the MTT solution was removed and 2-propanol was added to dissolve the formazan crystals metabolized by viable cells (Figure 2 C) under a continuous stirring in the shaker for 20 minutes. The optical density of the formazan solution was evaluated by a Tescan sunrise plate reader at 570 nm. The percentage of cell viability was expressed as the relative growth rate calculated by equation (2). Each result represents the mean viability ± standard error mean (SEM) of three independent experiments:


$$\text{Relative metabolic activity = }\frac{Absorbance\text{ of samples}}{Absorbance\text{ of control}}\times 100\ldots \;\ldots \;\ldots \text{(2)}$$

**Statistical analysis:** all the tests were performed as individual triplicate experiment (for in vitro tests) and quintuple experiment (for in vivo tests). The data were analyzed with the GraphPad Prism software version 5.0.3. All the data are expressed as mean ± standard error of mean (S.E.M. n = number of experiments). The statistical analyses were obtained by the analysis of variance (ANOVA), followed by the Bonferroni´s test where necessary. The confidence interval was set at 95% with a significance threshold of less than 5% (p<0.05).

**Ethical consideration:** all the animal experiments were carried out in accordance with the animal research: reporting of in vivo experiments (ARRIVE) guidelines and European Union (EU) directive 2010/63/EU for animal experiments and Cameroon National Ethic Committee (ref. FWIRB 00001954).

## Results

**Extraction yield of the leaves of *Vernonia amygdalina*:** the filtrate of the hydroalcoholic extract of the leaves of *V. amygdalina* obtained after maceration of 200 g of dried leaf powder for 24 hours in a water-ethanol mixture (70: 30) was concentrated with rotavapor at 45 °C and we obtained 12.4 g of plant extract, which gave a percentage of yield of 6.2%.

**Figure 3:**
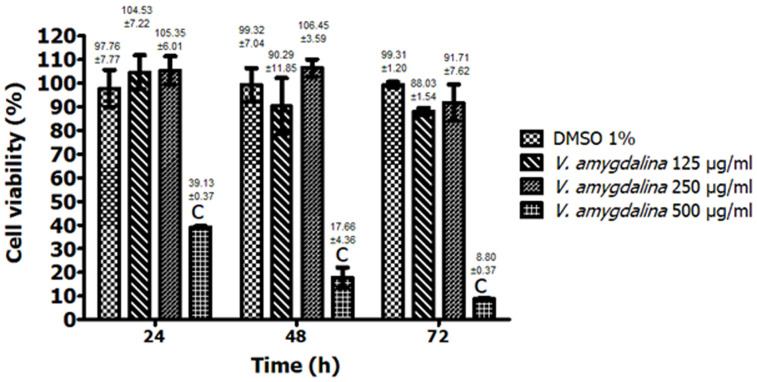
evaluation of the biocompatibility of the hydroalcoholic extract of *V. amygdalina* on rabbit fibroblasts; the results are expressed as mean ± S.E.M. (n=3) (cp<0.001), significant difference from the vehicle (1% DMSO in DMEM)

**In vitro biocompatibility:** the evaluation of the biocompatibility of HAEVa with rabbit fibroblasts is shown in Figure 3. This figure shows the percentage of cell viability following exposure of the fibroblasts to the different concentrations of HAEVa at 24, 48 and 72h respectively at concentrations of 125μg/ml (104.53 ± 2.22, 90.29 ± 11.85 and 88.03 ± 1.54, p>0.05) and 250μg/ml (105.35 ± 6.02, 106.45 ± 3.59 and 91.71 ± 7.62, p>0.05) compared to the vehicle (1% DMSO in DMEM) which presented cell viability rates (%) of 97.76 ± 7.77, 99.32 ± 7.04 and 99.31 ± 1.20 respectively at 24, 48 and 72h. This result revealed a highly significant cytotoxic activity of HAEVa (p<0.001) on rabbit fibroblasts only at the highest concentration of 500μg/ml (39.13 ± 0.37, 17.66 ± 4.36 and 8.80 ± 0.37 respectively at 24, 48 and 72h) compared to the vehicle. Interestingly, HAEVa in concentrations of 125 and 250 μg/ml was biocompatible with rabbit fibroblasts after 24, 48 and 72 hours of exposure.

**Figure 4:**
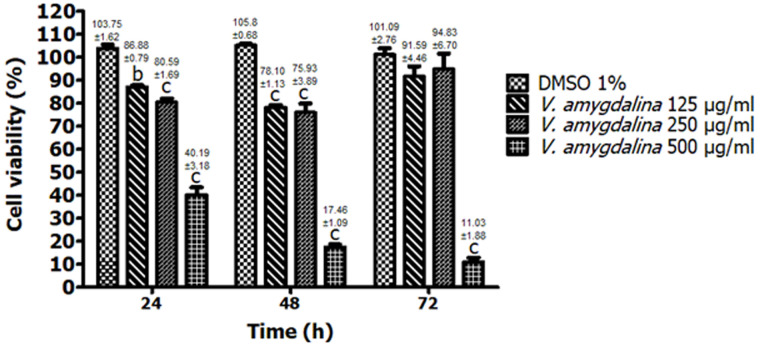
evaluation of the antiproliferative activity of the hydroalcoholic extract of *V. amygdalina* on human osteosarcoma MG-63; the results are expressed as mean ± S.E.M. (n=3), (cp<0.001), significant difference from the vehicle (1% DMSO in DMEM)

**In vitro antiproliferative activity:** Figure 4 shows the antiproliferative test on maligne MG-63 human osteosarcoma after contact with the different tested concentrations of HAEVa. Antiproliferative tests on MG-63 cancer cells revealed significant antiproliferative activity in a dose-dependent manner after 24 and 48 hours of exposure to plant extract at concentrations of 125μg/ml (86.88 ± 0.79, p<0.01 and 78.10 ± 1.13, p<0.001) and 250μg/ml (p<0.001, 80.59 ± 1.69 and 75.93 ± 3.89) compared to the vehicle which showed antiproliferative activity with cell viability percentages of 103.75 ± 1.62, 105.8 ± 0.68 and 101.09 ± 2.76 respectively at 24, 48 and 72h of exposure to Dimethyl sulfoxide (DMSO) 1% in MDEM. No significant difference (p>0.05) was observed in the percentage of cell viability at the 72nd hour of exposure of MG-63 human osteosarcoma cells to HAEVa at concentrations of 125 (91.59 ± 4.46) and 250μg/ml (94.83 ± 6.70). After 72 hours of exposure to the different tested concentrations of the plant extract, only the concentration of 500μg/ml induced highly significant antiproliferative activity (p<0.001) compared to the vehicle, with a percentage of cell viability of 40.19 ± 3.18, 17.46 ± 1.09 and 11.03 ± 1.88 respectively at 24, 48 and 72 hours of exposure to HAEVa.

**Figure 5:**
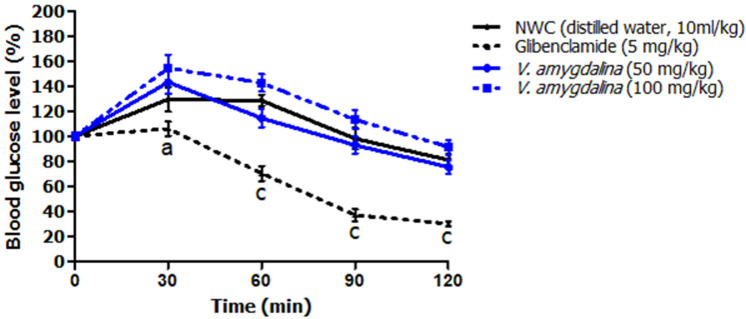
effects of hydroalcoholic extract of *V. amygdalina* on OGTT in normoglycemic rats; the results are expressed as mean ± S.E.M (n=5), (cp<0.001), significant difference from the CNN group

**Antihyperglycemic activity:** the result of the oral overload of a single dose of 3g/kg D-glucose in normoglycemic rats that received vehicle (negative control group), HAEVa at both doses tested (50 and 100mg/kg) and glibenclamide (positive control group) is shown in Figure 5. Thirty minutes after oral glucose overload, the negative control group had a postprandial blood sugar peak of 129.60 ± 9.83%, which decreased after 2 hours after oral glucose overload (81.20 ± 4.90%). However, rats that received HAEVa at both doses tested (50 and 100 mg/kg) did not significantly prevent (p>0.05) the postprandial blood glucose peak 30 minutes after gastric intubation of D-glucose compared to the normal negative control group (143.20 ± 9.10 and 154.40 ± 10.69% respectively at doses of 50 and 100mg/kg). Two hours after D-glucose administration, the plant extract stabilized postprandial blood glucose level at normal values at the two doses tested (75.40 ± 5.15 and 91.40 ± 6.05% respectively at doses of 50 and 100mg/kg) around the normal control group value. Unlike the plant extract, the group of animals that received glibenclamide, a reference insulin-secretor, significantly reduced the postprandial blood glucose peak 30 min after administration of D-glucose (106.00 ± 5.67%, p<0.05) as well as at the 60^th^, 90^th^ and 120^th^ (30.20 ± 2.17%) minute (p<0.001).

**Figure 6:**
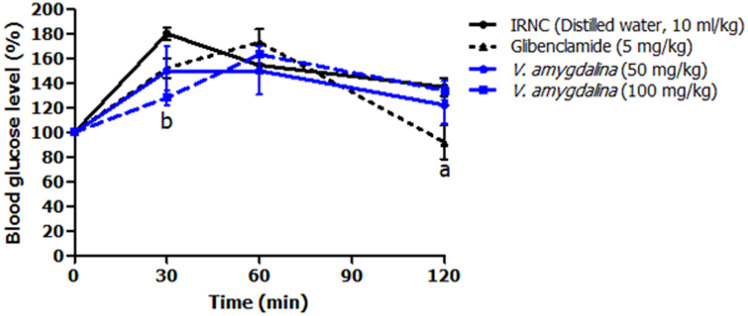
effects of hydroalcoholic extract of *V. amygdalina* on OGTT in insulin-resistant rats; the results are expressed as mean ± S.E.M (n=5), (ap<0.05; bp<0.01), significant difference from the CND group

Figure 6 shows the effect of HAEVa on postprandial blood glucose variation in insulin-resistant rats that experienced oral D-glucose overload after daily administration of the plant extract once daily for 14 days. It appears from this figure that after oral overload with D-glucose, the plant extract prevented the postprandial blood glucose peak (149.80 ± 20.19%, p>0.05 and 128.00 ± 6.02%, p<0.01) in insulin-resistant rats 30 min after administration of D-glucose at doses of 50 and 100 mg/kg respectively compared to the insulin-resistant negative control group (180.00 ± 5.09%). After 2h after oral D-glucose overload, HAEVa at 50mg/kg (122.20 ± 15.16%) had a greater effect in reducing postprandial blood glucose than 100mg/kg (134.00 ± 7.74%) and insulin-resistant negative control group (136.80 ± 7.33%). Neither dose tested showed a significant reduction (p>0.05) in blood glucose at the second hour after D-glucose administration compared to the insulin-resistant negative control group.

## Discussion

Extensive epidemiological studies suggest that certain types of cancers show a higher prevalence rate and a higher risk of mortality in a patient population suffering from diabetes mellitus. Sleeping cancer cells could be killed more readily than growing cancer cells in the body [13-15]. The hydroalcoholic extract of *V. amygdalina* exerted a highly significant cytotoxic activity (p<0.001) after 48 hours of exposure to concentrations of 125 and 250μg/ml on MG-63 cancer cells compared to the control group that was exposed to DMSO 1%. On the other hand, the concentration 500μg/ml of this plant extract highly induced (p<0.001) this cytotoxic activity on osteosarcoma MG-63 throughout the exposure period (72h) compared to the control group DMSO 1%. On the other hand, this plant extract exerted biocompatibility on rabbit primary dermal fibroblasts at concentrations of 125 and 250μg/ml (p>0.05) compared to the control group that was exposed to DMS0 solution 1% after 24, 48 and 72h of exposure. The concentration of 500μg/ml was highly cytotoxic (p<0.001) on rabbit fibroblasts throughout the period of exposure to the hydroalcoholic extract of *V. amygdalina* compared to the DMSO 1% control group. This suggests that this plant extract could activate tumor suppressor genes, such as the TP53 gene involved in many cancers in order to promote programmed cell death or slow its cycle, given that the mutation in the TP53 gene is associated with familial and sporadic forms of cancer [16]. It has been shown that the p53 protein is engaged in programmed cell death [17].

Repeated administration of dexamethasone in rats induced a significant increase in blood glucose level which caused insulin resistance. The treatment with hydroacoholic extract of *V. amygdalina* reduced significantly (p<0.01) the postprandial glucose level 30 min after administration of glucose when compared to the insulin-resistant negative control rats. The result obtained were similar to that of glibenclamide in the normoglycemic rats 30 min after administration of glucose when compared to the normal control group (p<0.05). OGTT is a well-accepted and frequently used assay to screen the antihyperglycemic activity of any hypoglycemic agent [18]. Glibenclamide is shown to stimulate insulin released by pancreatic β-cells. The bitter leaves of *V. amygdalina* could similarly stimulate the insulin released. This hypothesis is strengthened by previous study that the hydroalcoholic extract could potentiates the action of insulin by reducing insulin resistance observed in type 2 diabetes or could activate translocation of GLUT4 glucose transporters [12,19]. The hydroalcoholic extract of *V. amygdalina* prevented the postprandial blood glucose spike in a dose-dependent manner in dexamethasone-induced insulin resistant rats at the two doses tested (50 and 100 mg/kg), with a significant difference (p<0.01) only at the dose of 100 mg/kg compared to the insulin-resistant control group that received the vehicle. It has been reported that the leaves of *V. amygdalina* contains biflavonoids such as luteolin, luteolin 7-0-B-glucoside and luteolin 7-0-B-glucuronoside [20]. It is known that flavonoids are involved in the regulation of blood sugar, it is probable that the hypoglycemic activity of *V. amaygdalina* as reported in this study, may be a function of its rich flavonoid content [21].

It has also been reported that *V. amygdalina* simultaneously suppresses gluconeogenesis and potentiates glucose oxidation via the pentose phosphate pathway in streptozotocin-induced diabetic rats [22]. Hypoglycemic effects of bitter leaves of *V. amygdalina* have also been reported in experimental finishing chickens [23]. The antidiabetic efficacy of this plant in experimental finishing chickens found he percent reduction in blood glucose level was 14.30%, 22.90% and 28.60% for treatments with *V. amygdalina* inclusion rates of 5%, 10% and 15%, respectively. This suggests that the hydroalcoholic extract of *V. amygdalina* could have a mode of action similar to that of biguanides (metformin) by potentiating the action of insulin which has the effect of a decrease in the production of hepatic glucose as well as an increase in the transport of glucose in the muscle [24]. The ability of hydroalcoholic extract of *V. amygdalina* to stimulate glucose metabolism in muscle cells highlights the possible therapy of insulin resistance in the skeletal muscles [25].

## Conclusion

The results of this study suggest that HAEVa may up-regulate tumor suppressor genes to promote programmed cell death or down-regulate the cycle of MG-63 osteosarcoma. In addition, HAEVa prevents the postprandial blood sugar level in insulin-resistant rats, which suggests a decrease in the occurrence of cancer related to permanent hyperglycemia. These results justify the traditional use of *V. amygdalina* leaves in the treatment of cancer and diabetes mellitus in sub-Saharan Africa. Further studies would be possible to perform in vivo on various forms of cancers including osteosarcoma, in order to confirm the results obtained in vitro. However, given that the leaves of *V. amygdalina* are already consumed as food in SSA, it would be wise to propose an adequate formulation as a preventive measure against the onset of cancer or diabetes mellitus.

### What is known about this topic

- V. amygdalina is used in traditional medicine to treat diabetes mellitus and its complications;
- moreover, it has also been scientifically proven that this plant simultaneously suppresses gluconeogenesis and potentiates glucose oxidation via the pentose phosphate pathway in streptozotocin-induced diabetic rats (experimental type 1 diabetes mellitus).

### What this study adds

- The hydroalcoholic extract of V. amygdalina induces antiproliferative activity on human osteosarcoma MG-63 at concentrations of 125 and 250 μg/mL after 48 h of exposure; on the other hand, this plant extract is biocompatible at the same concentrations on rabbit dermal fibroblasts;
- in dexamethasone induced-insulin-resistant rats, the hydroalcoholic extract of V. amygdalina inhibits the postprandial blood glucose level in a dose-response manner (p>0.05 and p<0.01), respectively at doses of 50 and 100 mg/kg.
